# ﻿Higher predicted climate-change vulnerability for spring-dwelling freshwater biota

**DOI:** 10.3897/zookeys.1263.148253

**Published:** 2025-12-10

**Authors:** Mathias Kuemmerlen, Wolfram Graf, Johann Waringer, Simon Vitecek, Mladen Kučinić, Ana Previšić, Lujza Keresztes, Miklós Bálint, Steffen U. Pauls

**Affiliations:** 1 Department of River Ecology and Conservation, Senckenberg Research Institute and Natural History Museum Frankfurt, Clamecystr. 12, D-63571 Gelnhausen, Germany Senckenberg Research Institute and Natural History Museum Frankfurt Gelnhausen Germany; 2 Current address: Bundesamt für Naturschutz, Konstantinstraße 110, 53179 Bonn, Germany Bundesamt für Naturschutz Bonn Germany; 3 Institute of Hydrobiology and Aquatic Ecosystem Management, BOKU, University of Natural Resources and Life Sciences, Gregor Mendelstr. 33, 1180 Vienna, Austria BOKU, University of Natural Resources and Life Sciences Vienna Austria; 4 Department of Functional and Evolutionary Ecology, Division of Limnology, University of Vienna, Djerassiplatz 1, 1030 Vienna, Austria University of Vienna Vienna Austria; 5 Department of Biology, Faculty of Science, University of Zagreb, Rooseveltov trg 6, 10000 Zagreb, Croatia University of Zagreb Zagreb Croatia; 6 Center for Systems Biology, Biodiversity and Bioresources, Hungarian Department of Biology and Ecology, Babeş-Bolyai University, Clinicilor 5–7, 400006 Cluj-Napoca, Romania Babeş-Bolyai University Cluj-Napoca Romania; 7 Senckenberg Biodiversity and Climate Research Centre (BiK-F), Senckenberganlage 25, D-60325 Frankfurt am Main, Germany Senckenberg Biodiversity and Climate Research Centre (BiK-F) Frankfurt am Main Germany; 8 Senckenberg Research Institute and Natural History Museum Frankfurt, Senckenberganlage 25, D-60325 Frankfurt am Main, Germany Justus-Liebig-University Gießen Gießen Germany; 9 Institute of Insect Biotechnology, Justus-Liebig-University Gießen, Heinrich-Buff-Ring 26, 35392 Gießen, Germany Senckenberg Research Institute and Natural History Museum Frankfurt Frankfurt am Main Germany

**Keywords:** Caddisflies (Trichoptera), climate change, distribution change, Drusinae, endemism, range shifts, trait evolution

## Abstract

Environmental change threatens freshwater biodiversity through altered temperature and precipitation patterns. Available data is frequently insufficient to determine impacts at the species level leading to misinterpreted species’ vulnerability. Conversely, phylogenetic relationships, current distributions and ecological traits of the caddisfly subfamily Drusinae are well known. Thus, species distribution models (SDMs) were set up for 47 Drusinae species to assess individual and trait-specific climate change (CC) vulnerability. Species were grouped by larval feeding guild, stream zonation preference and level of endemism. Models were calibrated with predictors describing climate, topography and geology at a spatial resolution of 1 km^2^ and were projected for five general circulation models under four future climate scenarios. To limit dispersal, distribution projections were restricted to a maximum of 500 km until the year 2080. Relative predicted range change fluctuated between -100% and 197%, with extinction predicted for five species. Altitudinal shifts varied between -2% and +15%, with distribution centroids shifting between 28 km and 119 km. Our results identify stream zonation, a non-phylogenetic trait, as the best indicator of CC vulnerability. Furthermore, two important conclusions are highlighted: monitoring is best done at the species level while the biodiversity of springs and low order streams requires considerably more attention.

## ﻿Introduction

Direct anthropogenic impacts on the environment have been widely documented and discussed, but continue to magnify, causing significant biodiversity loss and ecosystem degradation across all biomes (Vitousek 1994; Sala et al. 2000; González-Orozco et al. 2016). Understanding the effects of such global environmental change on natural landscapes, habitats and organisms is paramount for the prevention and mitigation of further anthropogenic impacts. Ever-increasing and diversifying anthropogenic pressures have taken a major toll on freshwater ecosystems compared to other realms (Sala et al. 2000; WWF 2020). Threats include pollution, regulation of waterways through damming, overexploitation, water diversion, land-use and climate change (CC; Malmqvist and Rundle 2002; Reid et al. 2019). These impacts strongly affect freshwater biota which make up nearly 10% of global biodiversity and are restricted to habitats covering less than 1% of the earth’s surface (Strayer and Dudgeon 2010).

The potential effects of global environmental change are frequently forecasted through species distribution models (SDMs), based on future environmental scenarios (Elith and Leathwick 2009). Despite their rich biodiversity and high vulnerability, few such studies have been conducted for freshwater ecosystems. CC has been suggested to have significant impacts on the distribution and persistence of benthic macroinvertebrates in Europe, North America and Australia (Domisch et al. 2013; Bush et al. 2014; Shah et al. 2014; Pyne and Poff 2017; Theodoropoulos and Karaouzas 2021). However, these studies mostly rely on data at coarse taxonomic resolutions (i.e. genus or family level). Therefore, species-level research is urgently needed to appropriately infer the possible consequences of global environmental change, as well as to monitor the rate of biodiversity loss (Turak et al. 2017). Local extirpation or even extinction of taxa reduces biodiversity, alters community composition and can potentially modify ecosystem function with consequences for the stability of ecosystems and their services (Cardinale et al. 2012; Hooper et al. 2012; Hautier et al. 2015).

In freshwater ecosystems, for example, benthic macroinvertebrates influence energy flows and nutrient cycling (Covich et al. 1999). Therefore, it is of utmost importance to accurately determine the potential impact of future environmental conditions on different species’ distributions as a requisite to deduce possible consequences for ecological processes (Chapin et al. 2000; Woodward et al. 2010), particularly because much of the existing evidence in this regard stems from experimental work (Bärlocher et al. 2008; Friberg et al. 2009). In this context, species traits are of particular interest because they allow establishing links between individual taxa and their specific ecosystem functions (McGill et al. 2006; Poff et al. 2006), while indicating potential vulnerability to environmental change (Hershkovitz et al. 2015).

**Figure 1.**
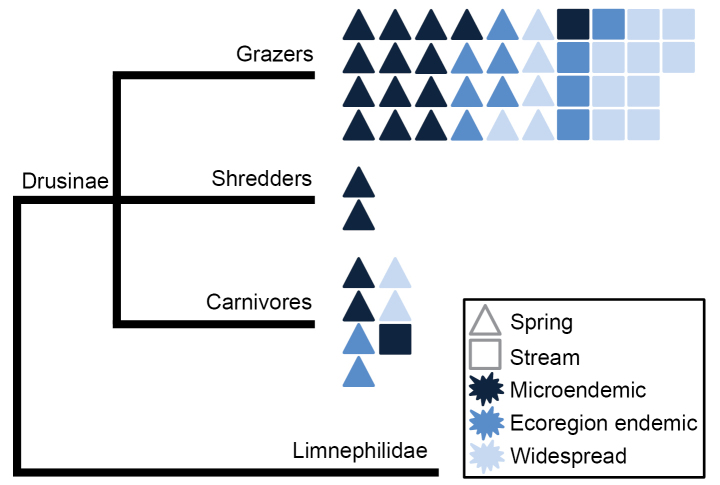
Phylogenetic tree of Drusinae caddisflies showing ecological traits: larval feeding guild, stream zonation preference and level of endemism. Modified from Pauls et al. (2008) and Vitecek et al. (2015a). Note that the genera of Drusinae are all either monotypic or paraphyletic. Thus, the currently valid generic nomenclature does not reflect natural evolutionary groupings and should be ignored when interpreting the results in an evolutionary or phylogenetic context.

In the present study, we projected the distributions of 47 caddisfly species of the Drusinae BANKS (1916) subfamily using SDMs and analysed their individual responses to expected future changes in climate. We account for ecological traits, as the effects of CC on modelled species’ distributions have shown to be highly variable in freshwater ecosystems (Domisch et al. 2013). Our analysis included the traits larval feeding guild, stream zonation preference (i.e. occurrence along the stream network) and degree of endemism to detect possible relationships with changing climatic conditions (Fig. 1; Bohle 1983; Waringer et al. 2007; Graf et al. 2008). These traits provide essential information on the functional roles of particular groups of species in freshwater ecosystems (e.g. processing of autochthonous organic matter by grazers) and their distribution in the stream network (e.g. springs or streams; Cardinale 2011). The high relevance of these traits is underlined by their application to determine the potential vulnerability of many aquatic insects to CC (Hering et al. 2009) and to create a climate change vulnerability score (CCVS; Hershkovitz et al. 2015).

The caddisfly subfamily Drusinae (Limnephilidae) is a clearly defined evolutionary group, with a well-studied phylogeny and abundant occurrence records at the species level (Pauls et al. 2008). In addition, the Drusinae (i) comprise three larval feeding strategies: grazers, shredders or carnivores; (ii) exhibit differential preferences for particular regions in the stream network: either springs or streams; and (iii) show various distribution patterns, ranging from microendemic species restricted to one single valley, to widespread species ranging across several European highlands and mountain ranges (Fig. 1). These characteristics renders them an optimal taxon to investigate CC effects on individual species, on ecological trait groups and ultimately, on ecosystem function (Wallace and Webster 1996). Moreover, some ecological traits such as larval feeding guild, are linked to distinct evolutionary clades while others, including river zonation preferences and level of endemism, are not (Fig. 1; Pauls et al. 2008; Vitecek et al. 2015a). This allows inferring whether CC vulnerability is embedded in the phylogenetic signal, or if it is unrelated to evolutionary history.

Predicted future climatic conditions in Europe foresee increased air temperatures ranging from +1.6 to +4.6 °C and an altered precipitation of -10 to +22% when comparing the time periods 1971–2000 and 2071–2100 (Jacob et al. 2014). Domisch et al. (2013) predicted considerable changes and shifts in suitable habitats for stream macroinvertebrates for the year 2080 under CC conditions, with endemic species experiencing the largest reductions in their range. The Drusinae are considered as highly vulnerable to environmental changes, especially CC, as they are found predominantly in Eurasian mountain ranges: from the Caucasus and the Iranian Highlands in the East, to the Iberian Peninsula on the West and from Scandinavia in the North to the Balkan Peninsula in the South (Dirnböck et al. 2013). In addition, many species are highly restricted (i.e. microendemics), a trait linked to CC vulnerability (Schwartz et al. 2006).

The goal of the present study was to use distribution predictions to evaluate the species-specific vulnerability of the Drusinae caddisflies to CC and, in a second step, identify which ecological traits are indicative of CC vulnerability. We evaluate three hypothesis: (i) that Drusinae caddisflies are highly vulnerable to CC, losing significant areas of their range and experiencing considerable altitudinal shifts (e.g. uphill) under future climate scenarios; (ii) that species grouped by differing ecological traits have similar distribution patterns (e.g. spring species have smaller and higher ranges than stream species); and (iii) that ecological trait groups with smaller ranges at higher elevations are more vulnerable to CC, with phylogenetic constraints playing a subordinate role in CC responses. We also compare the CC vulnerability stemming from the analysis of our distribution predictions with the CCVS, an existing classification regarding CC threats for individual species (Hershkovitz et al. 2015). Finally, we discuss potential phylogenetic signal of climate change vulnerability and its consequences to ecosystem functioning.

## ﻿Methods

To assure robust distribution predictions for freshwater biodiversity in general, and the Drusinae in particular, SDMs must consider environmental predictors that significantly contribute to shaping freshwater habitats (Domisch et al. 2015). Drusinae caddisflies prefer mostly small, cold-water streams and present very high levels of endemism, often being limited to spring brooks within a single mountain range (Graf et al. 2008; Previšić et al. 2009; Waringer et al. 2010). Accordingly, next to climate, we included geological predictors, as well as surrogate variables for hydrological discharge, flow velocity and solar irradiance. The model was set up for Europe, excluding the easternmost part, as well as the European portion of Turkey, because of limited availability of biological and environmental data.

### ﻿Biological and environmental data

Georeferenced occurrences of Drusinae caddisflies were taken from the Distribution Atlas of European Trichoptera (DAET; Schmidt-Kloiber et al. 2015) and checked individually for plausibility according to location and elevation by regional specialists on this subfamily (Waringer et al. 2016). The initial dataset contained 9,862 individual records of 66 species. Due to the large number of microendemic species, we attempted models for species with eight or more plausible occurrence sites, but not all of them yielded satisfactory results. Unsatisfactory results were generally the case for species with few occurrence records (< 12), which showed poor model performance (see definition below). Successful models were obtained for 47 species (Suppl. material 1).

Environmental predictors included nine topographical, geological and climatic variables, preselected based on several criteria: (i) low intercorrelation (r < |0.7|; Dormann et al. 2013); (ii) expert knowledge on the ecology and taxonomy of the group and (iii) variable importance in preliminary model runs. Topographical variables included slope, aspect (i.e. slope orientation) and flow accumulation (i.e. tributary upstream area), which were used as surrogates for flow velocity, exposure to the sunlight (i.e. solar irradiance) and catchment size, respectively (Domisch et al. 2015). The two former predictors were calculated from the digital elevation model (DEM) of the HYDRO1K database of the United States Geological Survey (USGS) using the software ArcGIS (ESRI, Redlands CA, USA), while the latter was merged from two different datasets: below 60° latitude from the flow accumulation stemming from the HydroSHEDS dataset (Lehner et al. 2008) and above 60° latitude from calculated flow accumulation based on the HYDRO1K DEM. Geology was incorporated as a categorical predictor with nine categories representing diverse parent materials (consolidated-clastic-sedimentary rocks, sedimentary rocks, igneous rocks, metamorphic rocks, unconsolidated deposits, unconsolidated glacial deposits, aeolian deposits, organic materials, no information) and taken from the European Soil Database (Panagos et al. 2012). As climatic predictors, both diurnal and annual temperature ranges, isothermality (daily to annual temperature range ratio), annual precipitation and precipitation seasonality (coefficient of variation indicating temporal distribution of rainfall) were chosen from the WorldClim dataset (Nix 1986; Hijmans et al. 2005).

To forecast distributions for the year 2080, models were projected for all scenarios of the fifth Assessment Report of the Intergovernmental Panel on Climate Change (AR5, IPCC), which are based on four greenhouse gas concentration trajectories known as Representative Concentration Pathways (RCP 2.6, 4.5, 6.0 and 8.5; IPCC 2013). The underlying data from regional climatic models of the EURO-CORDEX project for Europe, have been found to agree with observations and reanalyses for the main variables (Vautard et al. 2021).

RCPs are modelled predictions of global greenhouse gas (GHG) emissions that take socioeconomic drivers into account. Each RCP describes a possible pathway in the development of GHG emissions and atmospheric concentrations, based on different scenarios of air pollutant emissions and land use. RCP 2.6 assumed strict mitigation measures leading to significant reductions in GHG emissions, while RCP 8.5 allowed for very high GHG emissions. RCPs 4.5 and 6.0 were intermediate scenarios. The RCP pathways were implemented in various general circulation models (GCM), which results in different predictions of future climatic conditions. In the present study, five frequently implemented GCMs were projected for each RCP scenario (CSIRO Mk3.6.0, IPSL CM5A-LR, MRI CGCM3, NCAR CCSM4, NCC NorESM1-M). The future climate predictors were acquired from the Downscaled GCM Data Portal of the Consultative Group on International Agricultural Research (CGIAR) Research Program on Climate Change, Agriculture and Food Security (CCAFS; Ramirez and Jarvis 2008).

### ﻿Model set-up

The spatial resolution of the model was 1 km^2^ (30 arc-seconds), resulting in 10,649,788 raster grid cells in the extent considered. Models were calibrated with the R package biomod2 using three randomly selected pseudo-absence runs, five algorithms (GLM, GBM, CTA, ANN and MaxEnt) and two repetitions for each combination (Pauls et al. 2013; Schmitt et al. 2014). Thus, 30 models were calculated for each species, of which only those with a true skill statistic (TSS; Allouche et al. 2006) value above 0.6 were selected, weighted according to their TSS value and summarised in the final ensemble model. Single repetitions were cross-validated using an occurrence subset (30%) chosen randomly (Thuiller et al. 2009). Ensemble models were projected individually for present conditions and all four IPCC RCP scenarios. Final RCP scenario projections were assembled as a majority consensus from the five GCMs used (Domisch et al. 2013). To evaluate the models, we used the TSS indicator of model performance, as well as the area under curve (AUC, or receiver operating characteristic = ROC), sensitivity and specificity. Indicators of model performance and variable importance were averaged across the community and across groups when necessary for the further analysis.

### ﻿Further analysis

Present and future predicted distributions were first projected for all of Europe and later cropped using an area obtained by buffering the current known occurrences with a radius of 500 km. This buffer was assumed to roughly represent the maximum dispersal of Drusinae caddisflies that could be expected until the year 2080. This maximum dispersal distance was derived from the average dispersal velocity of 7.3 km per year determined for a caddisfly of the same family (Limnephilidae, *Potamophylax cingulatus*), during its invasion and expansion across Iceland in the last decades (Gíslason et al. 2015). Generally, caddisflies are considered to be poor dispersers (Dijkstra et al. 2014), and this empirical measurement is among the largest determined for adult aquatic insects (Kovats et al. 1996), making the buffer applied in our study, a realistic estimate at the upper end of possible rates of expansion. The buffered predictions were stacked for all Drusinae and for each ecological trait group to determine predicted richness, range changes, altitude and geographical shifts.

To identify differences between the current and each one of the RCP scenario distributions in terms of range changes or range shifts, we calculated the mean predicted range size, mean altitude (extracted from DEM) and the centroid of each predicted distribution. This information was also extracted for the ecological trait groups (i) larval feeding; (ii) river zonation preference; and (iii) level of endemism (Bohle 1983; Waringer et al. 2007; Graf et al. 2008). The feeding guilds of the Drusinae assessed in this study comprised grazers, carnivores and shredders (38, 7 and 2 species, respectively, Suppl. material 1). Because of the low number of shredders, only the two larger feeding groups were analysed. Concerning stream zonation preference, species known to inhabit predominantly springs (crenal) or streams (rhithral) were selected (32 and 15 species respectively). Species were classified into three levels of endemism, (i) microendemics: occurring in one single mountain range and within one ecoregion, (ii) ecoregion endemic: occurring in a single European freshwater ecoregion and (iii) widespread: present in two or more ecoregions (18, 12 and 17 species, respectively; Illies 1978). Classification criteria were mostly taken from Graf et al. (2008) as provided by the database www.freshwaterecology.info (Schmidt-Kloiber and Hering 2015) and either complemented or updated in a few cases with recently published (Previšić et al. 2013, 2014) and unpublished data (Suppl. material 1).

To assess the vulnerability of the Drusinae subfamily to CC, present and scenario projections were compared in terms of predicted range size and average altitude using paired Wilcoxon two-sample tests. To compare the present predictions between ecological trait groups, we used Wilcoxon two-sample tests. Paired Wilcoxon two-sample tests were used again to evaluate CC vulnerability within ecological trait groups by comparing present with scenario predictions. To confirm whether particular traits (e.g. microendemism) were truly good indicators of CC vulnerability, we tested whether their relative range changes, relative altitudinal shifts and geographic shifts differed from that of the complementary groups. Tests within groups always compared either grazers vs carnivores or spring species vs stream species. Microendemics and ecoregion endemics, however, were always compared to widespread species. For this purpose, Wilcoxon two-sample tests were used. Finally, a graphical comparison of the CCVS extracted from Hershkovitz et al. (2015) and the predicted range losses was made for each species.

## ﻿Results

Models performed very well on average, with mean values of 0.96 ± 0.06 (mean ± S.D.), 0.99 ± 0.01, 99.1 ± 2.0 and 96.7 ± 4.4 for TSS, AUC, sensitivity and specificity, respectively (Suppl. material 2).

**Figure 2.**
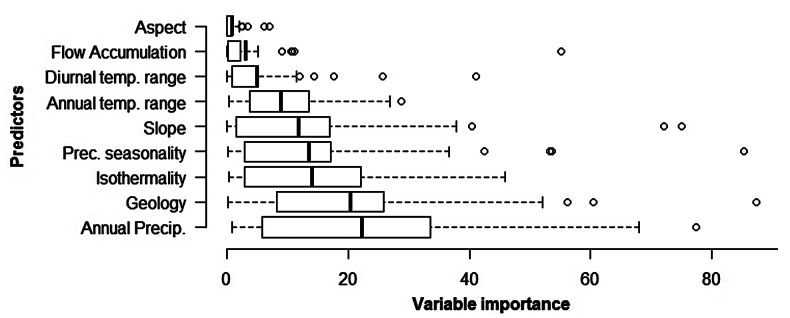
Variable importances of nine environmental predictors for 47 species.

Annual precipitation was, on average, the most important environmental predictor across all modelled species (mean 22% ± 19% S.D.; Fig. 2, Suppl. material 2), very closely followed by geology (20% ± 18%). Isothermality (14% ± 12%) and precipitation seasonality (14% ± 18%) were important predictions. Slope, annual temperature range and diurnal temperature range were of intermediate importance, while flow accumulation and aspect were the least important predictors. The variable importance of all predictors showed a wide range of values, indicating high variability among species.

### ﻿Present predictions

**Figure 3.**
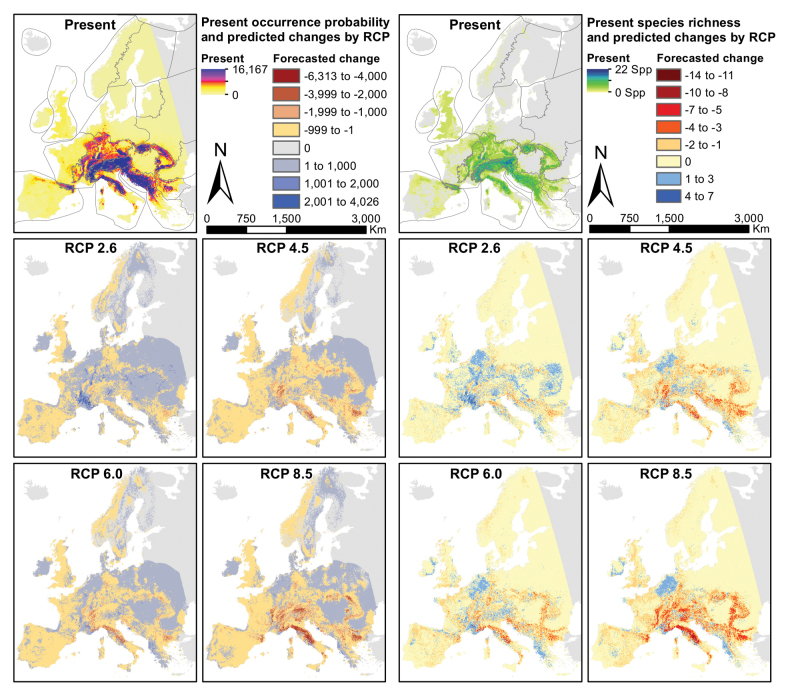
Stacked predictions for 47 species of the Drusinae, for the present projection (top) and four scenario forecasts (RCPs 2.6, 4,5, 6.0 and 8.5). The five panels on the left depict occurrence probability, the five panels on the right show predicted occurrence. Maps produced in R, v. 3.3.2 (https://www.r-project.org/), using data layers as described in the Methods section.

The highest stacked predicted probabilities of occurrence concentrate in the main European mountain ranges, such as the Pyrenees, the Alps, the Apennines, the Carpathians and the Dinaric Alps (Fig. 3). These coincide with predicted species richness: the Drusinae occur across most of Europe, but show high species richness in the aforementioned mountain ranges. Predicted range size varied widely, from 1,423 km^2^ for *Metanoea malickyi* covering two ecoregions, to 1,828,871 km^2^ for *Ecclisopteryx dalecarlica* distributed across 21 ecoregions. Present predicted range size did not differ within larval feeding guilds (Table 1; p > 0.05). Spring species, microendemics and ecoregion endemics, however, were predicted to have significantly smaller ranges than their complementary groups (stream species [p < 0.01] and widespread species [p < 0.001], respectively).

**Table 1.** Average absolute and relative changes in range size, altitude and shifts in space for feeding groups and zonation preferences, for present and four RCP scenario projections. Bold values indicate significant differences.

			Larval feeding guild				Stream zonation preference			
			Carnivores		Grazers		Spring species		Stream species	
	Scenario	Value	Average	SD	Average	SD	Average	SD	Average	SD
Range size (thousand km^2^)	Present	Abs.	266.8	299.3	322.2	468.4	**196.2^e^**	350.2	**526.4^e^**	528.4
	RCP 2.6	Abs.	292.6	331.5	328.7	505.5	200.7	372.4	545.5	583.8
	RCP 4.5	Abs.	**247.4^a^**	294.5	**259.8^c^**	417.9	**152.4^c^**	286.5	**452.6^a^**	508.4
	RCP 6.0	Abs.	265.6	298.4	**276.0^b^**	433.9	**166.4^a^**	304.2	472.5	520.1
	RCP 8.5	Abs.	**225.3^a^**	253.9	**228.6^c^**	379.6	**130.8^c^**	255.9	**408.2^b^**	461.6
Change in range size (%)	RCP 2.6	Rel.	-6.6	38.2	-15.8	38.2	-20.4	42.5	0.2	19.0
	RCP 4.5	Rel.	-32.1	34.6	-34.9	44.4	-37.4	48.8	-24.0	23.7
	RCP 6.0	Rel.	-15.8	36.2	-19.8	64.4	-19.5	70.9	-15.8	23.2
	RCP 8.5	Rel.	-29.2	41.2	-40.7	53.5	-46.6	52.5	-21.3	42.1
Mean altitude (m)	Present	Abs.	**1210.6^d^**	361.6	**945.5^d^**	378.4	**1109.5^e^**	428.5	**796.9^e^**	274.5
	RCP 2.6	Abs.	1186.6	355.6	958.1	403.0	1131.0	434.6	780.2	280.5
	RCP 4.5	Abs.	1231.0	469.7	**1027.5^c^**	453.5	**1227.6.0^c^**	485.7	819.3	327.8
	RCP 6.0	Abs.	1234.0	364.3	963.4	421.0	1146.9	455.8	807.9	313.5
	RCP 8.5	Abs.	1278.7	467.1	**1073.8^c^**	560.3	**1283.0^c^**	585.6	830.3	370.8
Altitudinal shift (%)	RCP 2.6	Rel.	-2.1	3.2	0.9	10.6	1.6	10.8	-2.3	6.0
	RCP 4.5	Rel.	-0.4	9.3	9.2	17.9	10.5	19.4	2.1	8.8
	RCP 6.0	Rel.	1.9	7.3	1.7	9.8	2.2	9.9	0.5	7.8
	RCP 8.5	Rel.	4.1	4.1	12.0	28.3	**14.7^d^**	31.1	**2.5^d^**	8.8
Geographical shift (km)	RCP 2.6	Abs.	28.3	26.2	33.7	26.0	31.3	27.5	32.7	22.6
	RCP 4.5	Abs.	54.5	56.8	75.0	60.7	66.5	78.1	78.1	40.7
	RCP 6.0	Abs.	34.0	17.1	65.2	62.4	61.7	65.5	55.8	37.9
	RCP 8.5	Abs.	**37.6^a^**	39.8	**114.5^a^**	91.6	103.1	99.3	96.2	67.1

^a^ significantly different from present prediction (p < 0.05) ^b^ significantly different from present prediction (p < 0.01) ^c^ significantly different from present prediction (p < 0.001) ^d^ significantly different within group (p < 0.05) ^e^ significantly different within group (p < 0.01) ^f^ significantly different within group (p < 0.001)

**Figure 4.**
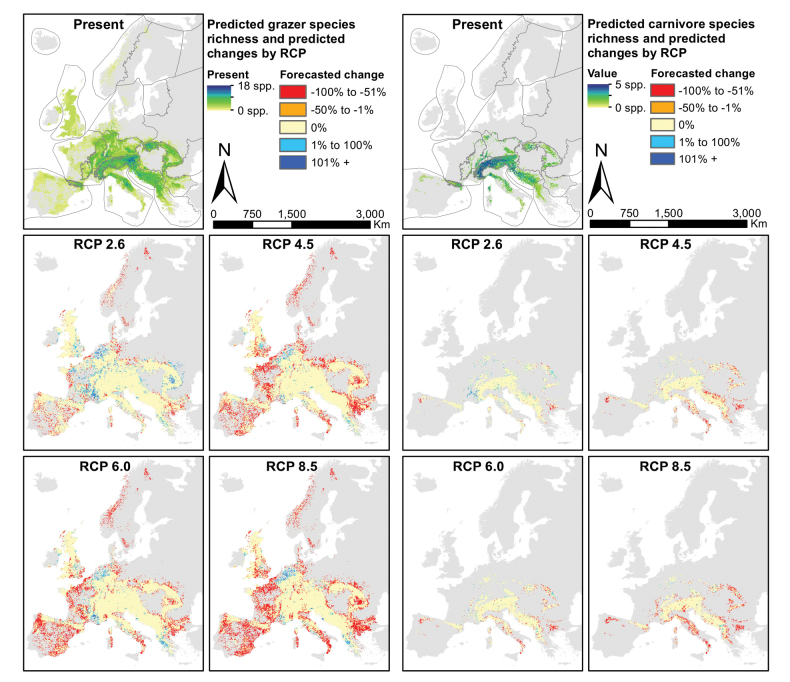
Species richness predictions for two feeding groups of the Drusinae, for the present projection (top) and four scenario forecasts (RCPs 2.6, 4,5, 6.0 and 8.5). The five panels on the left depict grazers, the five panels on the right show carnivores. Maps produced in R, v. 3.3.2 (https://www.r-project.org/), using data layers as described in the Methods section.

Modelled species were predicted to occur across a wide altitudinal gradient, from an average altitude of 427 m a.s.l. for *Drusus annulatus*, to 2,349 m a.s.l. for *D. nigrescens*. Carnivores, spring species, microendemics and ecoregion endemics were all predicted to occur, on average, at higher altitudes than their counterparts: grazers (p < 0.05, Fig. 4), stream species (p < 0.01) and widespread species (p < 0.01; p < 0.05), respectively.

### ﻿Scenario forecasts

Distribution predictions for individual species showed high heterogeneity in all four scenarios with some species gaining and some losing range. On average, however, the net effect was a range contraction across all modelled species, which was significant in all, but the RCP 2.6 scenario (p < 0.01 for RCP 6.0, p < 0.001 for RCPs 4.5 and 8.5; Table 2). In general terms, species richness was predicted to decline most strongly in the mountain ranges of the South such as the Apennines and the southern portions of the Pyrenees, as well as the Dinaric Alps (Fig. 3). Few extinctions were predicted under the future scenarios: one species in RCP 2.6 (*M. malickyi*), five in RCP 4.5 (*Drusus balcanicus*, *D. meridionalis*, *D. pallidus*, *D. spelaeus* and *M. malickyi*), three in RCP 6.0 (*D. balcanicus*, *D. spelaeus* and *M. malickyi*) and four in RCP 8.5 (*D. balcanicus*, *D. meridionalis*, *D. spelaeus* and *M. malickyi*). *Metanoea malickyi* was the only species predicted to become extinct in all scenarios.

**Table 2.** Average absolute and relative changes in range size, altitude and shifts in space for level of endemism and all Drusinae, for present and four RCP scenario projections. Bold values indicate significant differences.

			Level of endemism						All Drusinae	
			Microendemics		Ecoregion end.		Widespread			
	Scenario	Value	Average	SD	Average	SD	Average	SD	Average	SD
Range size (thousand km^2^)	Present	Abs.	**37.8^f^**	35.8	**162.7^f^**	144.5	**678.9^f^**	540.3	301.6	438.0
	RCP 2.6	Abs.	32.5	37.0	164.7	140.7	708.5	597.2	310.8	472.9
	RCP 4.5	Abs.	**23.7^c^**	28.0	**131.8^b^**	127.2	**568.1^b^**	505.1	**248.2^c^**	392.4
	RCP 6.0	Abs.	29.5	35.7	136.0	121.7	602.2	516.5	**264.1^b^**	406.8
	RCP 8.5	Abs.	**24.8^a^**	36.9	**103.9^b^**	103.2	**506.8^c^**	461.8	**219.3^c^**	355.1
Change in range size (%)	RCP 2.6	Rel.	-**33.4^d^**	47.4	-2.1	24.2	-**1.2^d^**	24.4	-13.8	37.7
	RCP 4.5	Rel.	-**48.4^d^**	49.1	-23.0	42.6	-**24.1^d^**	31.3	-33.1	42.6
	RCP 6.0	Rel.	-33.0	68.7	4.4	73.1	-18.8	30.5	-18.3	59.6
	RCP 8.5	Rel.	48.9	60.4	-37.1	44.7	-28.6	44.2	-38.6	50.8
Mean altitude (m)	Present	Abs.	**1047.2^d^**	393.5	**1249.8^e^**	471.9	**800.7^e,d^**	274.4	1009.8	410.4
	RCP 2.6	Abs.	1101.7	408.7	1226.8	490.7	**783.2^a^**	263.5	1016.6	422
	RCP 4.5	Abs.	1112.2	498.6	**1315.1^a^**	540.2	**893.7^c^**	334.0	**1081.8^c^**	474.9
	RCP 6.0	Abs.	1065.0	438.0	1280.3	510.0	826.0	285.8	1031.4	440.2
	RCP 8.5	Abs.	1155.3	601.0	**1465.2^b^**	617.9	**860.0^a^**	325.6	**1125.1^c^**	560.3
Altitudinal shift (%)	RCP 2.6	Rel.	4.5	13.0	-2.1	5.7	-2.0	6.2	0.4	9.6
	RCP 4.5	Rel.	4.7	12.7	4.3	7.9	12.0	22.9	7.5	16.8
	RCP 6.0	Rel.	-0.3	13.3	2.1	4.7	3.0	7.1	1.6	9.2
	RCP 8.5	Rel.	9.9	37.0	16.5	25.8	6.7	13.0	10.4	26.1
Geographical shift (km)	RCP 2.6	Abs.	31.0	31.3	27.4	14.1	35.6	26.8	31.8	25.8
	RCP 4.5	Abs.	**48.8^d^**	43.0	65.7	58.1	**90.7^d^**	65.4	70.6	58.7
	RCP 6.0	Abs.	55.8	55.6	61.3	80.0	62.0	40.8	59.7	57.2
	RCP 8.5	Abs.	**70.5^d^**	90.3	115.3	99.3	**115.3^d^**	77.6	100.7	88.6

^a^ significantly different from present prediction (p < 0.05) ^b^ significantly different from present prediction (p < 0.01) ^c^ significantly different from present prediction (p < 0.001) ^d^ significantly different within group (p < 0.05) ^e^ significantly different within group (p < 0.01) ^f^ significantly different within group (p < 0.001)

The average altitude based on all Drusinae predictions increased significantly in the RCP 4.5 and 8.5 scenarios (p < 0.001), indicating a general upward shift (Table 2). Altitudinal shifts ranged from -17.2% (downhill) for *D. franzressli* in RCP 2.6 to 93.6% (uphill) for *D. mixtus* in RCP 8.5.

Predicted range shifts across geographical space for all modeled species based on centroids were lowest in the RCP scenario 2.6, with values between 4.0 km and 125.3 km (Table 2). Shifts were predicted to increase significantly in all further scenarios (p < 0.01) when compared to RCP 2.6, reaching the maximum value in RCP 8.5 with 339.3 km. The average shift direction was Northwest, with predictions indicating range expansions into mountainous ecoregions such as the Alps, the Western and Central Highlands and the Hellenic western Balkans, but also into the Western and Central Plains (Fig. 3).

### ﻿Feeding groups

Predicted ranges size for carnivores decreased significantly in the RCP 4.5 and 8.5 scenarios (both p < 0.05, Table 1), while for grazers this was the case in RCP 4.5, 6.0 and 8.5 (p < 0.001, p < 0.01, p < 0.001 respectively; Table 1). The relative range loss for both groups followed a similar trend, but was not significant. Also, no significant change in average altitude was detected for carnivores, whereas grazers showed significant uphill shifts in the RCP scenarios 4.5 and 8.5 (both p < 0.001). However, no differences in relative altitudinal changes were detected between the two groups. Carnivores were predicted to shift significantly less in geographical space than grazers in the RCP scenario 8.5 (p < 0.001). Grazers lost range throughout Europe, while gaining new range in mountainous areas north of the Alps, such as the Central and Western Highlands, but also in the Hellenic Western Balkan and the Central and Western Plains (Fig. 4). Conversely, carnivores predominantly lost range, mostly in mountain ranges east and south of the Alps, in the Apennines, the Dinaric Western Balkan and the Carpathians. Relative number of extinctions were similar between grazers (4 of 38 spp. (10.5%): *D. balcanicus*, *D. pallidus*, *D. spelaeus* and *M. malickyi*) and carnivores (1 of 7 spp. (14.3%): *D. meridionalis*).

### ﻿Stream zonation

**Figure 5.**
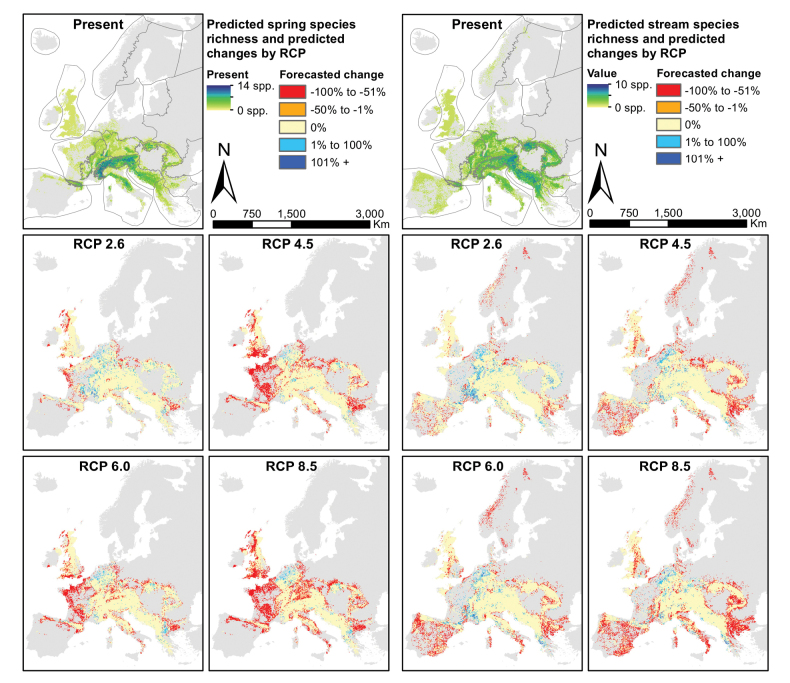
Species richness predictions for two stream zonation groups of the Drusinae, for the present projection (top) and four scenario forecasts (RCPs 2.6, 4,5, 6.0 and 8.5). The five panels on the left depict spring species, the five panels on the right show stream species. Maps produced in R, v. 3.3.2 (https://www.r-project.org/), using data layers as described in the Methods section.

Predicted ranges of spring species significantly decreased in RCP scenarios 4.5, 6.0 and 8.5 (p < 0.001, p < 0.05 and p < 0.001, respectively: Table 1), while those of stream species decreased only in RCP scenarios 4.5 and 8.5 (p < 0.05 and p < 0.01, respectively). In terms of relative range changes, no differences were observed between stream zonation preferences. Furthermore, significant uphill shifts were detected for spring species in RCP scenarios 4.5 and 8.5 (both p < 0.001), but none for stream species. The relative altitudinal shift of spring species is significantly stronger than that of stream species in RCP scenario 8.5 (p < 0.05). Similar shift distances in geographical space were predicted for spring and stream species. Spring species were forecasted chiefly to lose ranges in lowland areas such as the British Isles and the Western Plains, in mountainous areas like the Apennines, the Carpathians, the Western and Central Highlands, and to gain some range in the Alps and the Hellenic Western Balkans (Fig. 5). Predictions showed a roughly opposite pattern for stream species, which expanded their ranges in the Western and Central Highlands, while losing the most range in the Iberian Peninsula and the Eastern Balkans. All species predicted to become extinct are classified as spring species.

### ﻿Endemics

Predicted ranges decreased significantly in the RCP scenarios 4.5 and 8.5 for microendemics (p < 0.001 and p < 0.05, respectively; Table 2), for ecoregion endemics (both p < 0.01), and for widespread species (p < 0.01 and p < 0.001, respectively). Relative range changes in microendemics were significantly stronger than those of widespread species in RCP scenarios 2.6 and 4.5 (both p < 0.05). Significant altitudinal upward shifts were determined for the ecoregion endemics in RCP scenarios 4.5 and 8.5 (p < 0.5, p < 0.01, respectively) and for widespread species in RCP scenarios 2.6, 4.5 and 8.5 (p < 0.5, p < 0.001, p < 0.05, respectively), but not for microendemic species.

**Figure 6.**
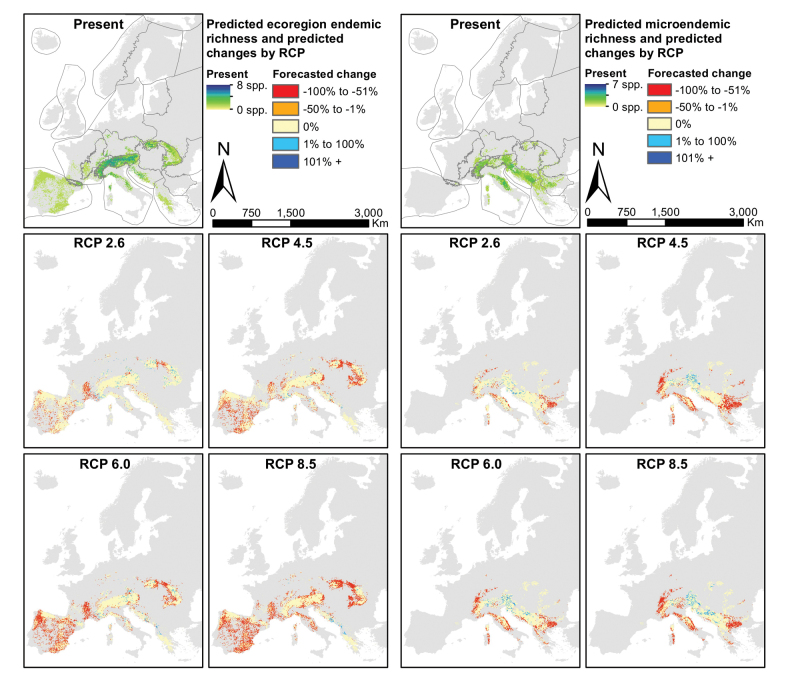
Species richness predictions for two levels of endemism of the Drusinae, for the present projection (top) and four scenario forecasts (RCPs 2.6, 4, 5, 6.0 and 8.5). The five panels on the left depict ecoregion endemics, the five panels on the right show microendemics. Maps produced in R, v. 3.3.2 (https://www.r-project.org/), using data layers as described in the Methods section.

Relative altitudinal shifts were similar within all levels of endemism. Moreover, microendemics experience a significantly smaller geographical shift than the widespread species in RCP scenarios 4.5 and 8.5 (both p < 0.05). Ecoregion endemics experience severe range losses in the Iberian Peninsula, Western Highlands, the Carpathians and the Eastern Balkans, but also range gains in mountain ranges such as the Dinaric Western Balkans (Fig. 6). Microendemics are also predicted to increase in richness in the Dinaric Western Balkans, remain stable in the Carpathians and decrease in the Apennines, the Western Highlands and the Eastern Balkans. All species predicted to become extinct are classified as microendemics.

### ﻿Climate change vulnerability

The vulnerability to CC estimated from SDMs based on future scenarios showed some important differences to those indicated by the climate change vulnerability score (CCVS, ranging from 0 = invulnerable, to 6 = highly vulnerable; Table 3) of Hershkovitz et al. (2015). In our predictions, most species lost range in at least two scenarios, which signals a reasonable vulnerability to CC. However, low CCVS scores (0 to 3) were assigned to 65% of the species included in our analysis, indicating low vulnerability. Moreover, of those species classified as invulnerable (CCVS = 0), all were predicted to lose range in at least one scenario, albeit the losses are predicted were comparatively low. In one case, the range lost was comparable to the range gained, indicating a range shift (*D. biguttatus*). Also, the five species predicted to become extinct in the present study, are classified with moderate to low CCVS values (*D. balcanicus* [4], *D. meridionalis* [4], *D. pallidus* [2], *D. spelaeus* [1] and *M. malickyi* [1]). In contrast, some species classified as vulnerable to CC with moderate to high CCVS such as *D. adustus* (4) and *E. asterix* (5), were predicted to gain range in all RCP scenarios.

**Table 3.** Climate change vulnerability for each species assessed by classifying predictions into one of eight classes of relative range change in the four RCP scenarios (2.6, 4.5, 6.0, 8.5) and counting them (scenario frequency). Colours indicate the frequency projections assigned to either positive (range gain) or negative (range loss) quartiles. The highest positive quartile includes predicted range gains above 100%. The climate change vulnerability score (CCVS) by Hershkovitz et al. (2015) is included for comparison, which ranges from 0 = invulnerable to 6 = highly vulnerable.

Species	Climate change vulnerability score	Range loss				Range gain			
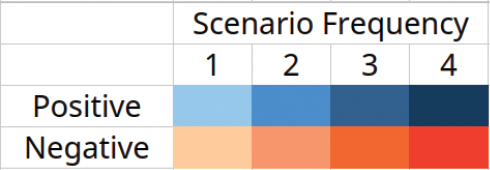		-100%	-75%	-50%	-25%	1%	26%	51%	76%
		to	to	to	to	to	to	to	to
		-76%	51%	26%	-1%	25%	50%	75%	100% +
*Anomalopterygella chauviniana*	0								
*Drusus biguttatus*	0								
*Drusus tenellus*	0								
*Ecclisopteryx dalecarlica*	0								
*Ecclisopteryx madida*	0								
*Drusus botosaneanui*	1								
*Drusus mixtus*	1								
*Drusus rectus*	1								
*Drusus spelaeus*	1								
*Ecclisopteryx guttulata*	1								
*Leptodrusus budtzi*	1								
*Metanoea malickyi*	1								
*Drusus annulatus*	2								
*Drusus bolivari*	2								
*Drusus brunneus*	2								
*Drusus chrysotus*	2								
*Drusus franzressli*	2								
*Drusus improvisus*	2								
*Drusus ingridae*	2								
*Drusus krusniki*	2								
*Drusus pallidus*	2								
*Drusus siveci*	2								
*Drusus trifidus*	2								
*Metanoea flavipennis*	2								
*Metanoea rhaetica*	2								
*Monocentra lepidoptera*	2								
*Drusus discolor*	3								
*Drusus graecus*	3								
*Drusus monticola*	3								
*Drusus popovi*	3								
*Drusus schmidi*	3								
*Cryptothrix nebulicola*	4								
*Drusus aprutiensis*	4								
*Drusus balcanicus*	4								
*Drusus camerinus*	4								
*Drusus carpathicus*	4								
*Drusus croaticus*	4								
*Drusus adustus*	4								
*Drusus melanchaetes*	4								
*Drusus meridionalis*	4								
*Drusus nigrescens*	4								
*Drusus romanicus*	4								
*Drusus alpinus*	5								
*Drusus franzi*	5								
*Drusus muelleri*	5								
*Ecclisopteryx asterix*	5								
*Ecclisopteryx keroveci*	NA								

## ﻿Discussion

Distributions for widespread species, as well as for very restricted ones, were predicted with reasonable accuracy. Thus, we assume our model setup to appropriately describe the distributions of species with very specific habitat requirements and high endemism levels. Precipitation was an important predictor for describing species’ distributions, suggesting a strong link to discharge patterns driven by climate. Discharge and other hydrological regime components are among the most important factors determining the distribution of freshwater biodiversity at multiple scales, such as the extent used in this study (Poff and Zimmerman 2010), a fact that has also been substantiated in other SDMs (Kuemmerlen et al. 2015). Surprisingly, geology played an important role for all species, rather than only for those with special habitat preferences (e.g. karst springs). For five spring species (*D. carpathicus*, *D. discolor*, *D. graecus*, *D. schmidi* and *D. trifidus*) geology even accounted for most of the variation in the model (> 50%), which is remarkable, considering geology is only rarely implemented as an environmental predictor in general (Richards et al. 1997), or in SDMs (Kuemmerlen et al. 2016).

Average predictions for all Drusinae show clear range losses and uphill movements, suggesting significant range changes in the three most severe scenarios (RCP 4.5, 6.0, 8.5) and altitudinal shifts in two of these (RCP 4.5, 8.5). Accordingly, significant changes in Drusinae distributions may be expected if greenhouse gas emissions are not reduced in the near future and CC continues along the current trend. The magnitude of predicted range losses from the present study was lower than that determined by Sauer et al. (2011) but similar to those reported by Domisch et al. (2013). The high variability observed in projected range changes (e.g. for microendemics) are also comparable to observed changes in the flora of different European mountain tops that were related to various regional CC effects (Pauli et al. 2012).

Although we observed geographic variation in range changes, predicted species richness losses and range contractions were mostly projected south of the Alps, where climatic conditions are generally warmer and drier. This coincides with species-loss predictions in North America (Pyne and Poff 2017). Our predictions resulted in a maximum annual dispersal velocity of approximately 4.8 km per year for *D. pallidus* in the Balkan Peninsula, considerably slower than the 7.3 km per year determined for the recent expansion of the caddisfly *P. cingulatus* in Iceland (Gíslason et al. 2015). The geographical shifts predicted in the present study can thus be considered realistic and conservative by comparison.

Overall, our projections confirm our first hypothesis that Drusinae caddisflies are highly vulnerable to CC and give rise to serious concerns on how general community composition in freshwater ecosystems could be altered under future climatic conditions. Even for species with lower predicted vulnerability to CC, the generalised tendency is to disperse into higher elevations at the expense of decreasing their predicted range sizes. This dispersal is likely to lead to novel biological interactions between species with similar ecological traits, which adds to stress induced by CC, resulting in an elevated extinction risk (Hooper et al. 2012). Our model predicted five species to become extinct, but under the influence of continuous and increasing climatic changes over a longer time frame, additional species could be lost. Moreover, extinction risks reported here are similar to other estimates (Thomas et al. 2004). Beyond species extinction events, changes in distribution patterns can threaten individual populations with extirpation, putting at risk the subsistence of intraspecific lineages (Taubmann et al. 2011; Bálint et al. 2011; Schmitt et al. 2014). All five species predicted to become extinct, are microendemic spring-dwellers of middle elevations. This resembles the results from previous SDM predictions based on bioclimatic variables (Domisch et al. 2011) and could be a consequence of the combined impact of climate change and morphological alterations on low mountain range streams. Preserving genetic diversity is at the core of species conservation, as it is required for species to adapt and evolve under changing environmental conditions (Pauls et al. 2013; Stark et al. 2025). Therefore, beyond focusing conservation efforts on particular traits, efforts should also be made to preserve as many individual populations of species as possible. For this purpose, appropriate conservation measures would have to be developed and implemented locally.

Carnivorous Drusinae, although few in number, occur at higher elevations than grazers according to present projections, but the size of their predicted ranges remained similar. Moreover, range losses for carnivores are only projected in two future CC scenarios and altitude shifts were not projected. Conversely, grazers showed some vulnerability to climate change through range losses in three scenarios, altitudinal shifts in two and geographic shifts in one scenario. These results indicate that larval feeding behaviour is not a useful ecological trait to assess CC vulnerability, because (i) carnivores and grazers do not sufficiently differ in terms of range losses and (ii) carnivores, as the arguably most threatened group because of their higher average altitude, show no altitudinal shifts and lesser geographical shifts than grazers under future scenarios. Because larval feeding types did not clearly differ in CC sensitivity, our ability to draw conclusions regarding ecosystem functions (e.g. autochthonous organic matter processing by grazers) is limited. The causes for this poor differentiation may be twofold: the comparatively low number of carnivores limiting statistical power (unrelated to any sampling artefacts; see Fig. 1) and the fact that most carnivore species are also classified as spring species, which indicates that this trait may better reflect CC sensitivity than feeding type or other phylogenetically constrained traits.

In the present projection, spring species were predicted to have significantly smaller ranges and at higher altitudes than stream species. Spring species were also predicted to lose range and experience uphill shifts in more RCP scenarios than stream species. Conversely, range losses in stream species are only projected in two RCP scenarios. Also, stream species are not predicted to experience altitudinal shifts, indicating lower CC vulnerability. The fact that spring species shift in altitude is particularly important because of the difficulty to track suitable climatic conditions within their habitat (i.e. along the stream network). As spring species inhabit exclusively springs and spring-brooks, they cannot disperse further upstream, while downstream aquatic dispersal requires traversing unsuitable habitats. These species are thus stuck in a ‘dead end’ and their survival will depend on tracking suitable habitats through ‘out-of-network’ (i.e. overland) dispersal. This kind of dispersal has been shown to be reduced beyond distances of 10–20 km for several species of *Drusus* (Previšić et al. 2009; 2014; Geismar et al. 2015). Stream species, on the other hand, exhibited a lower-than-average predicted altitude shift and are much more likely to be successful at dispersing through the stream network, both upstream and downstream. From our study, stream zonation preference emerges as a good ecological trait to identify CC vulnerability.

Range predictions suggest micro- and ecoregion endemics to have smaller ranges at higher altitudes than widespread species. Microendemism would initially imply a very high vulnerability to CC as such species lose range in greater proportions than widespread species, even in the two least severe RCP scenarios. Interestingly, neither further range losses nor altitudinal shifts increase with the severity of the scenarios in microendemics, while both increase for ecoregion endemics. The CC effects on relative range losses differentiated from those shown by widespread species, but in the two lower intensity scenarios. Moreover, widespread species experience altitudinal shifts more frequently than micro- or ecoregion endemics. The initial strong negative trend of climate change on the distribution of microendemics, does not continue to increase with the severity of the RCP scenarios. This could result from the relatively large dispersal capacity assumed, allowing any species to track climate across long distances in the modelled predictions. The projections of microendemics also result in a wide variety of reactions to CC across species and scenarios. For example, relative range changes in *D. croaticus* vary between +157.2% and -4.4% depending on the RCP scenario, while those in *D. siveci* are projected to vary from +36.5% to -49.8%. Therefore, level of endemism is likely not an appropriate ecological trait to gauge CC vulnerability in an SDM approach because projections may be misleading and need to be assessed carefully species by species.

Regarding our trait-based hypotheses, we can summarise that stream zonation is a relevant indicator for CC vulnerability because their predictions consistently exhibited negative influences of CC on their projected distribution patterns. The results also show that there are no evolutionary constraints on the reaction of species to climate change, as there is no signal for common climate change reactions among the three main evolutionary lineages that align with feeding group in Drusinae and as a result feeding groups appear to be poor predictors of climate change sensitivity.

We evaluated distribution predictions under *a posteriori* consideration of ecological traits and observed that changes in predicted distributions are strongly related to stream zonation preference. Conversely, previous studies have estimated climate change vulnerability directly from traits assigned to species, assuming homogenous responses between ecological trait groups (Hershkovitz et al. 2015). However, our results indicate that only some of the traits used in the CCVS are clear indicators of range changes potentially induced by CC. As CC vulnerability of species varies in response to many different extrinsic and intrinsic factors, a simple and universal approach would be most correct if restricted to a single ecological trait which is a clearly proven indicator. For this purpose, stream zonation preference was informative in Drusinae. Further, species-specific models are likely to be more accurate than a generalised approach that considers many species to be similar in traits, thus likely ignoring species-specific responses.

A holistic approach including indices based on known species traits and changes in predicted occurrences (e.g. via SDM) could be a suitable solution integrating information provided by both approaches. As the number of SDMs predicting future distributions continues to increase – stimulated by specific calls for an expansion in biodiversity monitoring (Pereira et al. 2013; Schmeller et al. 2018) – distribution predictions can be more readily combined with trait-based approaches. Furthermore, SDMs are likely to continue being vital components in CC studies, as they deliver key criteria used in the IUCN Red List classification, while providing information for planning, management, monitoring and decision making (Rodrigues et al. 2006).

Distribution predictions for Drusinae caddisflies showed a general vulnerability to CC, but responses were highly variable among species. Stream zonation was associated with CC vulnerability, but without links to particular evolutionary lineages of Drusinae (Fig. 1). Thus, we found no evidence of phylogenetic dependency in the responses to CC, and the expectation that congeners or otherwise closely related species may react similarly through a constraint based on phylogenetic niche and trait conservatism, is not warranted. This outcome is particularly important in freshwater systems, where worldwide most data are generated through biomonitoring surveys based on higher rank classifications (e.g. genus or family level). Ultimately, monitoring should aim at fine taxonomic resolution, ideally at the species level (but see Houghton 2025). Lumping together species into higher taxonomic units will certainly lead to incomplete knowledge on the biodiversity of sites, but may also fail to detect the extent of CC impacts on freshwater communities (Bálint et al. 2011).

More generally, additional anthropogenic impairments to freshwater ecosystems such as habitat destruction, land-use changes, pollution, eutrophication, water abstraction and structural modification, among many others, are not considered here, although they are omnipresent (Schmalz et al. 2015). It is certain that these additional factors will exacerbate the effects of CC, further reducing distributions and preventing shifts to new suitable habitats (Brook et al. 2008). In fact, the impact of land use change on stream macroinvertebrates has been observed to exceed that of CC (Kuemmerlen et al. 2015), a further reason to consider the results presented here as conservative. Here, grazers are partially predicted to experience range expansions into the Western and the Northern Plains, however, these areas are unlikely to be suitable as they are of intensive agricultural use and watersheds are impacted by a large variety of anthropogenic pressures. Thus, our predictions need to be assessed bearing in mind that global environmental change comprises more than just CC. In the specific case of the Drusinae for example, small-scale hydropower development poses a serious additional threat likely to destroy significant stretches of suitable habitats in medium and small mountain streams of the Balkan region, where species richness and endemism are highest, but also elsewhere in Europe (Zarfl et al. 2015; Vitecek et al. 2015a).

## ﻿Conclusions

Our study on Drusinae caddisflies can serve as an example of the possible outcomes of changing climate conditions on freshwater biodiversity. The need for increased CC research on freshwater ecosystems becomes evident from two main points. First, several species of Drusinae have only been described recently in Europe (Vitecek et al. 2015b; Ibrahimi et al. 2016), a region considered as one of the best-studied globally regarding caddisflies. This exemplifies how efforts to fully assess the diversity and distribution of this taxonomic group may turn out to be a race against time, let alone determining the levels of intraspecific variability. Second, at small spatial scales, biodiversity loss has profound impacts on ecosystems (i) reducing the efficiency with which communities perform ecological processes (Covich et al. 1999; Cardinale 2011), (ii) lowering productivity (Hooper et al. 2012), (iii) reducing stability (Chapin et al. 2000) and (iv) becoming a relevant factor of ecosystem change in itself, matching other drivers of global environmental change such as CC (Hooper et al. 2012). Our analysis shows a general tendency towards biodiversity loss by CC, particularly for spring species. Paradoxically, springs belong to the *aqua incognita* of freshwater science, as they are repeatedly overlooked in monitoring schemes driven by policy such as the European Water Framework Directive (Bishop et al. 2008; Kuemmerlen et al. 2016). This, besides the high vulnerability of spring biodiversity to CC, highlights the increased attention they require. The detection of early signs of CC impact can be improved if monitoring fully considers spring dwelling species and their habitats.

## ﻿Additional information

### Use of AI

No use of AI was reported.

### Author ORCIDs

Mathias Kuemmerlen https://orcid.org/0000-0003-1362-3701

Wolfram Graf https://orcid.org/0000-0001-6559-0644

Johann Waringer https://orcid.org/0000-0002-0114-1636

Simon Vitecek https://orcid.org/0000-0002-7637-563X

Mladen Kučinić https://orcid.org/0000-0003-2756-2305

Ana Previšić https://orcid.org/0000-0002-0332-7522

Lujza Keresztes https://orcid.org/0000-0002-4609-6496

Miklós Bálint https://orcid.org/0000-0003-0499-8536

Steffen U. Pauls https://orcid.org/0000-0002-6451-3425

### Data availability

The datasets generated during and analysed during the current study are available from the corresponding author on reasonable request. Occurrence data for the Drusinae caddisflies are available upon request from the Distribution Atlas of European Trichoptera (DAET) (https://doi.org/10.15504/fmj.2015.9). Environmental predictor data is freely available from the cited sources. The R package used to build the SDMs, *biomod2* is accessible through the CRAN repository.

## Supplementary materials

Supplementary material 1 List of species modeled, with code, number of occurrences used in the model, classifications according to feeding group, endemism and stream zonation, as well as climate vulnerability score (CCVS) Data type docx https://binary.pensoft.net/file/1487251This dataset is made available under the Open Database License (http://opendatacommons.org/licenses/odbl/1.0/). The Open Database License (ODbL) is a license agreement intended to allow users to freely share, modify, and use this Dataset while maintaining this same freedom for others, provided that the original source and author(s) are credited.Mathias Kuemmerlen, Wolfram Graf, Johann Waringer, Simon Vitecek, Mladen Kučinić, Ana Previšić, Lujza Keresztes, Miklós Bálint, Steffen U. Pauls

Supplementary material 2 Model performance indicators and variable importance by species Data type docx https://binary.pensoft.net/file/1487252This dataset is made available under the Open Database License (http://opendatacommons.org/licenses/odbl/1.0/). The Open Database License (ODbL) is a license agreement intended to allow users to freely share, modify, and use this Dataset while maintaining this same freedom for others, provided that the original source and author(s) are credited.Mathias Kuemmerlen, Wolfram Graf, Johann Waringer, Simon Vitecek, Mladen Kučinić, Ana Previšić, Lujza Keresztes, Miklós Bálint, Steffen U. Pauls
