# Supplementary material for: ﻿Higher predicted climate-change vulnerability for spring-dwelling freshwater biota
Source: Zookeys. 2025 Dec 10;1263:289–315. doi: 10.3897/zookeys.1263.148253 (PMC12712645; doi:10.3897/zookeys.1263.148253)
Supplement: Supplementary material 1 — List of species modeled, with code, number of occurrences used in the model, classifications according to feeding group, endemism and stream zonation, as well as climate vulnerability score (CCVS) [file zookeys-1263-289_article-148253__-s001.docx]

# Supplementary material

**Higher predicted climate-change vulnerability for spring-dwelling freshwater biota**

Mathias Kuemmerlen*^1,9^, Wolfram Graf^2^, Johann Waringer^3^, Simon Vitecek^3^, Mladen Kučinić^4^, Ana Previšić^4^, Lujza Keresztes^5^, Miklós Bálint^6^, Steffen U. Pauls^6,7,8^

^1^ Senckenberg Research Institute and Natural History Museum, Frankfurt, Department of River Ecology and Conservation, Clamecystr. 12, D-63571 Gelnhausen, Germany;

^2^ University of Natural Resources and Life Sciences, Institute of Hydrobiology and Aquatic Ecosystem Management, Max-Emanuel-Straße 17, 1180Vienna; [wolfram.graf@boku.ac.at](mailto:wolfram.graf@boku.ac.at)

^3^ University of Vienna, Department of Limnology and Bio-Oceanography, Althanstrasse 14, A-1090 Vienna, Austria, [johann.waringer@univie.ac.at](mailto:johann.waringer@univie.ac.at), [simon.vitecek@univie.ac.at](mailto:simon.vitecek@univie.ac.at)

^4^ University of Zagreb, Faculty of Science, Department of Biology, Rooseveltov trg 6, 10000 Zagreb, Croatia; [mladen.kucinic@biol.pmf.hr](mailto:mladen.kucinic@biol.pmf.hr), [ana.previsic@biol.pmf.hr](mailto:ana.previsic@biol.pmf.hr)

^5^ Babeş-Bolyai University, Hungarian Department of Biology and Ecology, Center for Systems Biology, Biodiversity and Bioresources, Clinicilor 5–7, 400006 Cluj-Napoca, Romania; [lujza.keresztes@ubbcluj.ro](mailto:lujza.keresztes@ubbcluj.ro)

^6^ Senckenberg Biodiversity and Climate Research Centre (BiK-F), Aquatic Evolutionary Ecology, Senckenberganlage 25, D-60325 Frankfurt am Main, Germany, [steffen.pauls@senckenberg.de](mailto:steffen.pauls@senckenberg.de), [miklos.balint@senckenberg.de](mailto:miklos.balint@senckenberg.de)

^7^ Senckenberg Research Institute and Natural History Museum Frankfurt, Section Entomology III, Senckenberganlage 25, D-60325 Frankfurt am Main, Germany,

^8^ Institute of Insect Biotechnology, Justus-Liebig-University Gießen, Heinrich-Buff-Ring 26, 35392 Gießen, Germany

^9^ Current address: Bundesamt für Naturschutz, Konstantinstraße 110, 53179 Bonn, Germany; Mathias.Kuemmerlen@bfn.de

*Corresponding author

**Supplementary material 1, Table** List of species modeled, with code, number of occurrences used in the model, classifications according to feeding group, endemism and stream zonation, as well as climate vulnerability score (CCVS). Data from Graf *et al*. ^27^, except those in **bold**, taken from recent studies (see text).

| **Taxon** | **Code** | **Occurrences** | **Feeding group** | **Stream zonation** | **Ecoregion endemic** | **Micro-endemic** | **CCVS** |
| --- | --- | --- | --- | --- | --- | --- | --- |
| Anomalopterygella chauviniana | ano_cha | 304 | grazer | rhithral | No | No | 0 |
| Cryptothrix nebulicola | cry_neb | 113 | carnivore | crenal | Yes | No | 4 |
| Drusus adustus | dru_des | 27 | grazer | crenal | Yes | No | 4 |
| Drusus alpinus | dru_alp | 21 | shredder | crenal | Yes | Yes | 5 |
| Drusus annulatus | dru_ann | 1,443 | grazer | crenal | No | No | 2 |
| Drusus aprutiensis | dru_apr | 10 | grazer | crenal | Yes | Yes | 4 |
| Drusus balcanicus | dru_bal | 9 | grazer | **crenal** | Yes | Yes | 4 |
| Drusus biguttatus | dru_big | 307 | grazer | rhithral | No | No | 0 |
| Drusus bolivari | dru_bol | 41 | grazer | rhithral | Yes | No | 2 |
| Drusus botosaneanui | dru_bot | 75 | grazer | rhithral | No | No | 1 |
| Drusus brunneus | dru_bru | 87 | grazer | rhithral | Yes | No | 2 |
| Drusus camerinus | dru_cam | 13 | grazer | crenal | Yes | Yes | 4 |
| Drusus carpathicus | dru_car | 22 | grazer | crenal | Yes | No | 4 |
| Drusus chrysotus | dru_chr | 179 | carnivore | crenal | No | No | 2 |
| Drusus croaticus | dru_cro | 26 | grazer | crenal | Yes | Yes | 4 |
| Drusus discolor | dru_dis | 515 | carnivore | crenal | No | No | 3 |
| Drusus franzi | dru_fra | 19 | shredder | crenal | Yes | Yes | 5 |
| Drusus franzressli | dru_fzi | 11 | grazer | crenal | Yes | No | 2 |
| Drusus graecus | dru_gra | 10 | grazer | crenal | Yes | No | 3 |
| Drusus improvisus | dru_imp | 32 | grazer | crenal | No | Yes | 2 |
| Drusus ingridae | dru_ing | 12 | grazer | crenal | No | No | 2 |
| Drusus krusniki | dru_kru | 19 | grazer | **crenal** | Yes | Yes | 2 |
| Drusus melanchaetes | dru_mel | 68 | grazer | crenal | Yes | No | 4 |
| Drusus meridionalis | dru_mer | 9 | carnivore | crenal | Yes | **Yes** | 4 |
| Drusus mixtus | dru_mix | 33 | grazer | crenal | No | Yes | 1 |
| Drusus monticola | dru_mon | 142 | grazer | crenal | No | No | 3 |
| Drusus muelleri | dru_mue | 33 | carnivore | crenal | Yes | Yes | 5 |
| Drusus nigrescens | dru_nig | 42 | grazer | crenal | Yes | No | 4 |
| Drusus pallidus | dru_pal | 12 | grazer | crenal | Yes | Yes | 2 |
| Drusus popovi | dru_pop | 9 | grazer | **crenal** | Yes | Yes | 3 |
| Drusus rectus | dru_rec | 69 | grazer | crenal | No | No | 1 |
| Drusus romanicus | dru_rom | 12 | carnivore | crenal | Yes | No | 4 |
| Drusus schmidi | dru_sch | 20 | grazer | **rhithra**l | **No** | **No** | 3 |
| Drusus siveci | dru_siv | 9 | carnivore | **rhithral** | Yes | Yes | 2 |
| Drusus spelaeus | dru_spe | 8 | grazer | crenal | Yes | Yes | 1 |
| Drusus tenellus | dru_ten | 34 | grazer | rhithral | No | No | 0 |
| Drusus trifidus | dru_tri | 118 | grazer | crenal | No | No | 2 |
| Ecclisopteryx asterix | ecc_ast | 24 | grazer | crenal | Yes | Yes | 5 |
| Ecclisopteryx dalecarlica | ecc_dal | 438 | grazer | rhithral | No | No | 0 |
| Ecclisopteryx guttulata | ecc_gut | 268 | grazer | rhithral | No | No | 1 |
| Ecclisopteryx keroveci | ecc_ker | 9 | grazer | **rhithral** | No | **No** | NA |
| Ecclisopteryx madida | ecc_mad | 272 | grazer | rhithral | No | No | 0 |
| Leptodrusus budtzi | lep_bud | 27 | grazer | rhithral | No | Yes | 1 |
| Metanoea flavipennis | met_fla | 69 | grazer | rhithral | Yes | No | 2 |
| Metanoea malickyi | met_mal | 8 | grazer | crenal | No | Yes | 1 |
| Metanoea rhaetica | met_rha | 178 | grazer | rhithral | Yes | No | 2 |
| Monocentra lepidoptera | mon_lep | 48 | grazer | **crenal** | No | No | 2 |
