# Supplementary material for: ﻿Higher predicted climate-change vulnerability for spring-dwelling freshwater biota
Source: Zookeys. 2025 Dec 10;1263:289–315. doi: 10.3897/zookeys.1263.148253 (PMC12712645; doi:10.3897/zookeys.1263.148253)
Supplement: Supplementary material 2 — Model performance indicators and variable importance by species [file zookeys-1263-289_article-148253__-s002.docx]

# Supplementary material 2

**Higher predicted climate-change vulnerability for spring-dwelling freshwater biota**

Mathias Kuemmerlen*^1,9^, Wolfram Graf^2^, Johann Waringer^3^, Simon Vitecek^3^, Mladen Kučinić^4^, Ana Previšić^4^, Lujza Keresztes^5^, Miklós Bálint^6^, Steffen U. Pauls^6,7,8^

^1^ Senckenberg Research Institute and Natural History Museum, Frankfurt, Department of River Ecology and Conservation, Clamecystr. 12, D-63571 Gelnhausen, Germany;

^2^ University of Natural Resources and Life Sciences, Institute of Hydrobiology and Aquatic Ecosystem Management, Max-Emanuel-Straße 17, 1180Vienna; [wolfram.graf@boku.ac.at](mailto:wolfram.graf@boku.ac.at)

^3^ University of Vienna, Department of Limnology and Bio-Oceanography, Althanstrasse 14, A-1090 Vienna, Austria, [johann.waringer@univie.ac.at](mailto:johann.waringer@univie.ac.at), [simon.vitecek@univie.ac.at](mailto:simon.vitecek@univie.ac.at)

^4^ University of Zagreb, Faculty of Science, Department of Biology, Rooseveltov trg 6, 10000 Zagreb, Croatia; [mladen.kucinic@biol.pmf.hr](mailto:mladen.kucinic@biol.pmf.hr), [ana.previsic@biol.pmf.hr](mailto:ana.previsic@biol.pmf.hr)

^5^ Babeş-Bolyai University, Hungarian Department of Biology and Ecology, Center for Systems Biology, Biodiversity and Bioresources, Clinicilor 5–7, 400006 Cluj-Napoca, Romania; [lujza.keresztes@ubbcluj.ro](mailto:lujza.keresztes@ubbcluj.ro)

^6^ Senckenberg Biodiversity and Climate Research Centre (BiK-F), Aquatic Evolutionary Ecology, Senckenberganlage 25, D-60325 Frankfurt am Main, Germany, [steffen.pauls@senckenberg.de](mailto:steffen.pauls@senckenberg.de), [miklos.balint@senckenberg.de](mailto:miklos.balint@senckenberg.de)

^7^ Senckenberg Research Institute and Natural History Museum Frankfurt, Section Entomology III, Senckenberganlage 25, D-60325 Frankfurt am Main, Germany,

^8^ Institute of Insect Biotechnology, Justus-Liebig-University Gießen, Heinrich-Buff-Ring 26, 35392 Gießen, Germany

^9^ Current address: Bundesamt für Naturschutz, Konstantinstraße 110, 53179 Bonn, Germany; Mathias.Kuemmerlen@bfn.de

*Corresponding author

**Supplementary material 2, Table** Model performance indicators and variable importance by species
